# Feeling stiffness in the back: a protective perceptual inference in chronic back pain

**DOI:** 10.1038/s41598-017-09429-1

**Published:** 2017-08-29

**Authors:** Tasha R. Stanton, G. Lorimer Moseley, Arnold Y. L. Wong, Gregory N. Kawchuk

**Affiliations:** 1 The Sansom Institute for Health Research, School of Health Sciences & PainAdelaide Consortium, The University of South Australia, Adelaide, SA Australia; 2 Neuroscience Research Australia, Sydney, NSW Australia; 3 Department of Physical Therapy, Faculty of Rehabilitation Medicine, The University of Alberta, Edmonton, Alberta Canada; 4 Department of Rehabilitation Sciences, Faculty of Health and Social Sciences, The Hong Kong Polytechnic University, Hong Kong Special Administration Region, Hong Kong, China

## Abstract

Does *feeling* back stiffness actually reflect *having* a stiff back? This research interrogates the long-held question of what informs our subjective experiences of bodily state. We propose a new hypothesis: *feelings* of back stiffness are a protective perceptual construct, rather than reflecting biomechanical properties of the back. This has far-reaching implications for treatment of pain/stiffness but also for our understanding of bodily feelings. Over three experiments, we challenge the prevailing view by showing that *feeling* stiff does not relate to objective spinal measures of stiffness and objective back stiffness does not differ between those who report *feeling* stiff and those who do not. Rather, those who report *feeling* stiff exhibit self-protective responses: they significantly overestimate force applied to their spine, yet are better at detecting changes in this force than those who do not report *feeling* stiff. This perceptual error can be manipulated: providing auditory input in synchrony to forces applied to the spine modulates prediction accuracy in both groups, without altering actual stiffness, demonstrating that feeling stiff is a multisensory perceptual inference consistent with protection. Together, this presents a compelling argument against the prevailing view that *feeling* stiff is an isomorphic marker of the biomechanical characteristics of the back.

## Introduction

Bodily feelings constitute a fundamental aspect of self-awareness and provide critical homeostatic functions – e.g., feeling cold makes one seek warmth^1^; feeling pain makes one seek protection^2^; feeling parched makes one drink^3^. We assume that these bodily feelings reflect the biological state of our body tissues – a ‘read-out’, so to speak, of somatosensory and visceral input – particularly when the feeling is *located somewhere* in the body, as it is for pain or stiffness. There is growing evidence for pain however, that it is highly modulated by a wide range of cognitive and contextual variables^4, 5^. For example, visually manipulating the perceived size of one’s hand alters the pain experienced in experimental contexts^6^ and during movement of a chronically painful limb^7^, and illuminating a blue or red light in synchrony with delivering a noxious cold stimulus can transform the feeling evoked from uncomfortably cold to painfully hot^5^.

That somatic input triggered by physiological responses is important in cognitive processes – so-called ‘embodied cognition’ – has recently been countered with the idea that bodily awareness (e.g., our sense of bodily ownership) might directly modulate physiological regulation of body tissue in an anatomically specific way^8, 9^. Indeed, we now know that the sense of bodily ownership, the felt location, and the anthropometric characteristics of our body parts, are tightly linked to their physiological regulation in a bi-directional manner^7, 10^. Extensive human data from both healthy and diseased populations have led to the proposal of the cortical body matrix theory^11^, the predictions of which are yielding new developments in our understanding and treatment of some pathological pain conditions^12^.

One feeling that is almost universally experienced, which impacts on our daily lives^13, 14^, but which remains relatively ignored, is feeling stiff. Feeling joint stiffness is unanimously attributed to actually having stiff joints. Take for example having a ‘stiff back’. We assume that this feeling reflects altered force-response profiles of the tissues in our back (objective biomechanical stiffness). But does it? A feeling of joint stiffness has been shown to remain despite loss of the limb^15^ suggesting that the bodily feeling of stiffness can be independent to the biological state of the tissues.

Feeling stiff is a significant predictor of disability^16^ and is a primary target of interventions for many musculoskeletal conditions including back pain, arthritis, and neck pain. Worldwide, the most burdensome of these conditions is low back pain (LBP). Affecting ~10% of the world’s population (~632 million people), chronic LBP is also the leading cause of disability^17^. Despite increasing healthcare expenditure, clinical outcomes remain sub-optimal^18^, spurring a call to re-examine basic mechanisms associated with LBP and its symptoms^19^, most obviously stiffness.

Here we describe a series of experiments, the results of which compel us to rethink what it means to feel stiff, what determines how stiff we feel, and what ecological value feeling stiff might have. First, we aimed to establish whether feeling back stiffness is related to (i) actual stiffness of the back as reflected in force-response profiles, and (ii) a manifestation of actual stiffness that would offer a protective behavioural advantage – the ability to judge the magnitude (and change) of applied force to the back. Specifically, Study 1a interrogated the prevailing view that feelings of stiffness reflect objective biomechanical back stiffness. We hypothesised that if this theory is correct, subjective and objective back stiffness measures would closely relate, and people who feel back stiffness would objectively have a stiffer spine than those who do not. Study 1b evaluated whether people with feelings of back stiffness show protective perceptual responses – do they differ from those without feelings of stiffness at estimating how much force was applied to the spine and at detecting change in this force? We hypothesised that they would overestimate force, but be more sensitive to changes in force, suggestive of a protective profile.

Second, we hypothesised that feeling stiff should be modulated by sensory cues that are unrelated to actual back stiffness. To investigate this, we exploited the brain’s predilection for cross-modal integration by synchronising mechanical loading of the back with two different auditory signals – the sound of a ‘creaky door’ and a gentle ‘whooshing’ sound. Given the common pairing of sounds (grinding, popping) with movement of a stiff back, we anticipated that pairing sound with force applied to the spine would provide ecologically valid information that may alter protective perceptual responses. Specifically, Study 2 evaluated the empirical support for our new theory that feeling stiffness in the back represents a perceptual construct, which could be modified by additional body-relevant sensory information, such as sound. We hypothesised that sound would modify back perception and that this modification would be enhanced in those that report feeling back stiffness.

Overall, we postulate that feeling back stiffness represents a protective, perceptual inference: a bodily feeling created to provide the critical homeostatic function of preventing movement and potential re-injury. We do so based on the bi-directional, intimate relationship between perception and movement: when we move, we constantly receive sensory feedback, as a result of the movement, that informs our perception of body position, but we also perceive things in line with how we will act^20^. For example, people underestimate the distance from their body to a pain-relieving switch, but only when they are able to reach for it^20^. The following series of experiments comprehensively interrogate both the prevailing view that feeling back stiffness equates to having objective back stiffness (Study 1a) and our new hypothesis of stiffness as a protective perceptual inference (Study 1b and Study 2).

## Results

We recruited 15 people with chronic LBP who also reported having chronic *feelings* of back stiffness and 15 age- and gender-matched healthy controls without LBP or feelings of back stiffness. Table 1 presents their demographic and clinical information.

**Table 1:** Demographic and baseline questionnaire data.

Demographic variables	Study 1a and 1b			Study 2		
	CLBP & stiffness (n = 15)	Healthy controls (n = 15)	p-value	CLBP & stiffness (n = 10)	Healthy controls (n = 10)	p-value
Age (years)	27.1 (8.3)	28.9 (8.3)	0.54	23.4 (4.8)	25.7 (5.4)	0.33
Gender	11M, 4F	10M, 5F	0.69	7M, 3F	6M, 4F	0.64
Height (cm)	176.5 (11.1)	170.2 (11.1)	0.14	176.0 (11.2)	170.5 (13.5)	0.33
Weight (kg)	74.5 (16.5)	73.5 (15.3)	0.85	73.1 (16.5)	71.3 (13.3)	0.80
Current pain	34.3 (15.5)	1.2 (2.2)	<0.001	16.5 (18.4)	0.6 (1.6)	0.02
Avg pain last 48 hrs	36.7 (16.8)	0.6 (1.4)	<0.001	23.0 (17.7)	0.6 (1.6)	0.003
Duration of pain (years)	6.4 (5.1)	n/a	n/a	6.2	n/a	n/a
Current stiffness	43.0 (17.2)	2.0 (2.4)	<0.001	20.8 (16.8)	1.2 (2.1)	0.005
Avg stiffness last 48 hrs	41.3 (19.3)	2.3 (3.7)	<0.001	26.8 (19.2)	1.7 (3.3)	0.003
Body drawing (count)						
• Complete/accurate	2	7	0.046	1	4	0.12
• Incomplete/inaccurate	13	8		9	6	
Modified ODQ	31.2 (15.7)	1.3 (2.5)	<0.001	22.0 (9.7)	0.4 (0.8)	<0.001
Back Beliefs Questionnaire:						
Inevitability	29.9 (6.6)	29.9 (5.5)	0.77	20.3 (16.0)	20.9 (15.6)	0.74
State STAI-short form:						
Pre-testing	11.0 (4.0)	7.9 (1.7)	0.01	10.7 (4.1)	8.3 (2.1)	0.13
Post-testing	9.9 (3.4)	7.6 (1.6)	0.03	10.4 (3.7)	7.7 (1.9)	0.06

All values are mean (SD) unless otherwise specified. Pain and stiffness measures rated using a 0–100 numerical rating scale (NRS), where higher values represent more pain/stiffness. Body image drawings of the back assessed perception of the back^32^ and were coded as either complete/accurate or incomplete (missing parts of the back) or inaccurate (large deviations in drawing of the back). Modified Oswestry Disability Questionnaire (ODQ) has a minimum score of 0 and a total possible score of 100, higher scores represent higher levels of disability^67^. Back Beliefs Questionnaire has a minimum score of 9 and maximum score of 45 where lower scores represent increased negativity in back beliefs^68^. Spielberg State-Trait Anxiety Inventory (STAI) short form has a minimum score of 6 and a maximum score of 24; higher scores indicate higher levels of state anxiety^69^.

### Study 1a. *Feeling* stiff does not equate to *being* stiff in chronic LBP

**Figure 1:**
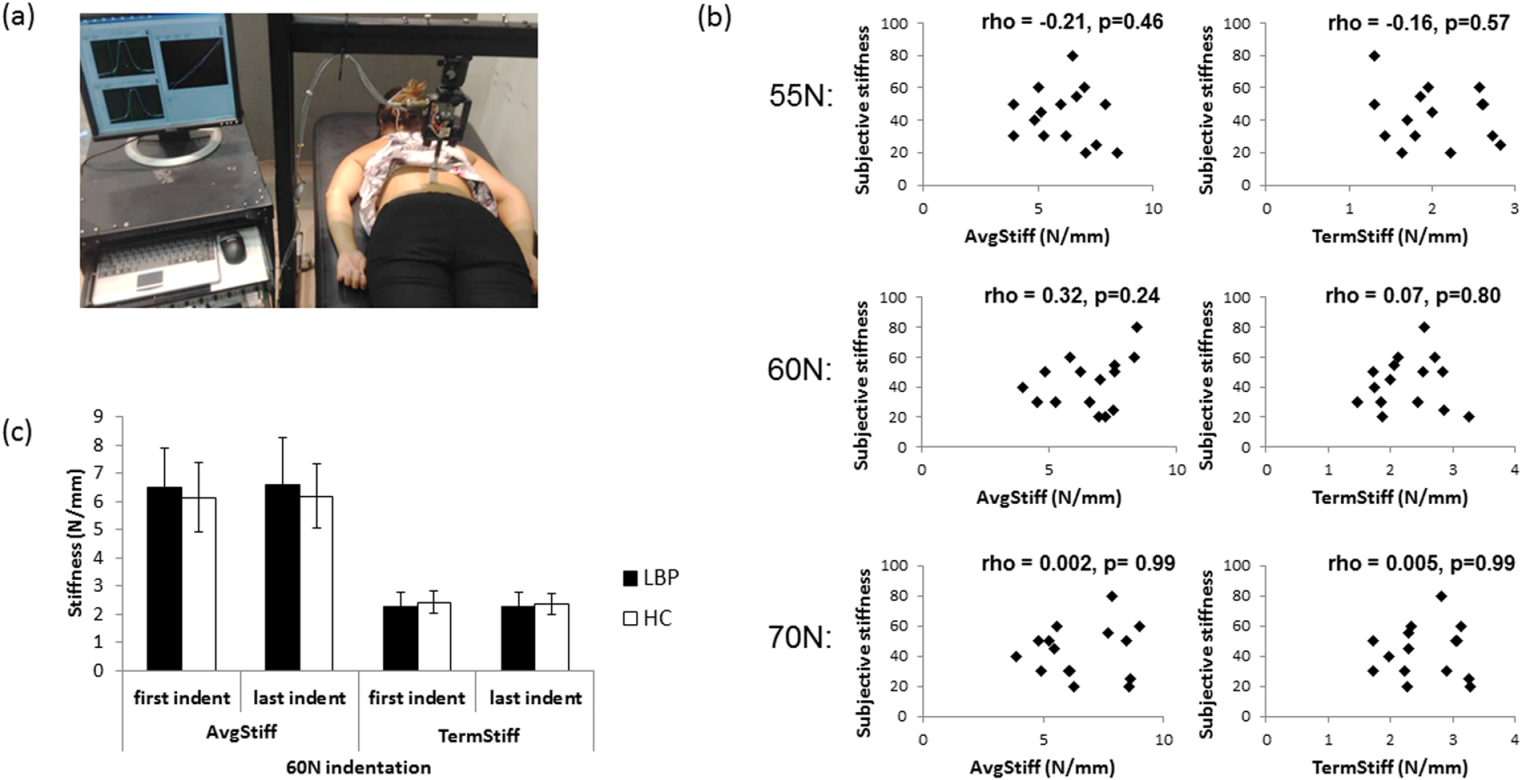
Study 1a results. (**a**) Picture of the indentor and participant set-up (**b**). Scatterplots depicting the correlation between feelings of stiffness and objective measures of stiffness in people with chronic back pain and stiffness (**c**). Mean and standard error for objective spinal stiffness measures at 60 N in healthy control participants and participants with chronic back pain and stiffness. Abbreviations: AvgStiff = Average stiffness; TermStiff = Terminal stiffness.

Study 1a used an established, customised device, validated in humans^21^, that provides a standardised force to the spine, measuring displacement of the application probe (Fig. 1a). Objective stiffness measures are calculated by considering this force–displacement relationship (Average Stiffness [AvgStiff] and Terminal Stiffness [TermStiff]; see *Methods*). We established how stiff participants’ backs ‘*felt*’ (subjective/perceived stiffness; 0–100 numerical rating scale [NRS], ranging from “not stiff at all” to “most stiff imaginable”) and how stiff their backs objectively were (customised device).

To determine if *feelings* of stiffness correspond to how stiff the back actually is, we used Spearman’s rho. We found no significant relationships between perceived and actual stiffness at any applied force level (55 N, 60 N, 70 N) or any stiffness measure (AvgStiff or TermStiff) in people with LBP and stiffness (p = 0.24–0.99; Fig. 1b). We were unable to investigate this relationship in healthy controls due to insufficient variability (8 of the 15 controls did not report feeling any back stiffness [rating of 0 on the 100-point NRS] and the remainder all reported stiffness of 5).

Second, we tested if objective measures of stiffness differed between those who reported feeling LBP and stiffness and those who reported they did not (n = 30). We considered objective stiffness measures at the start and end of testing to evaluate for any between-group differences in biomechanical spine properties secondary to repeated tissue loading (factor: ‘time’). There were no significant differences between groups in either of the objective spinal stiffness measures at any applied force level (Fig. 1c). Specifically, at 60 N (±1 N), the repeated measures MANOVA found no significant between-group difference for objective back stiffness measures (AvgStiff and TermStiff) over time (F_2,27_ = 1.04, p = 0.37, partial η^2^ = 0.072). Univariate test results from this analysis (stiffness measure specific) are presented in Table 2 and support the lack of effect of group, time or group x time interaction. A sensitivity analysis, evaluating only participants who received 60 N (n = 22), confirmed the above findings (Supplementary Table S1). Additionally, a MANOVA confirmed that there was no difference between groups (n = 30) for AvgStiff and TermStiff at 55 N (F_2,27_ = 2.45, p = 0.11, partial η^2^ = 0.153) and at 70 N (F_2,27_ = 0.98, p = 0.39, partial η^2^ = 0.068) suggesting that this lack of objective stiffness difference holds across the applied force spectrum (See Table 2 for univariate analyses results; Supplementary Figure S1). Using these validated methods, feeling stiffness in one’s back was not related to the biomechanical properties of the spine.

**Table 2:** Univariate results for objective spinal stiffness - Study 1a.

Factor	Test statistic	p-value	Effect size
AvgStiff at 60 N - Univariate results of repeated measures MANOVA			
Group	F_1,28_ = 0.60	0.44	partial η^2^ = 0.021
Time	F_1,28_ = 0.34	0.56	partial η^2^ = 0.012
Group x Time	F_1,28_ = 0.019	0.89	partial η^2^ = 0.001
*TermStiff at 60 N - Univariate results of repeated measures MANOVA*			
Group	F_1,28_ = 0.53	0.47	partial η^2^ = 0.019
Time	F_1,28_ = 1.86	0.18	partial η^2^ = 0.062
Group x Time	F_1,28_ = 1.16	0.21	partial η^2^ = 0.055
*Stiffness at 55 N - Univariate results of MANOVA*			
AvgStiff: Group	t_2,28_ = −0.082	0.94	d = 0.029
TermStiff: Group	t_2,28_ = 1.67	0.11	d = 0.61
*Stiffness at 70 N – Univariate results of MANOVA*			
AvgStiff: Group	t_2,28_ = 0.18	0.86	d = 0.062
TermStiff: Group	t_2,28_ = 1.03	0.31	d = 0.38

AvgStiff = average stiffness (objective measure); TermStiff = terminal stiffness (objective measure). Group = chronic low back pain and stiffness versus healthy controls; Time: first and last indentation at 60 N (unable to assess the effect of ‘time’ for 55 N and 70 N because very few participants received these force values twice).

### Study 1b. People with chronic LBP and stiffness demonstrate a protective profile: they overestimate force, but are better at detecting changes in force

Study 1b used established force magnitude estimation^22^ and difference detection^23^ tasks. Participants (n = 30) were advised that they would receive indentation forces between 50 N and 70 N to their back; these forces were applied in pairs (indentations 30 seconds apart; see below for assessment). Participants were asked to estimate, as accurately as possible, the magnitude of force delivered (see *Methods* for training procedure). Estimation errors were calculated for forces applied at 60 N (equal opportunity to over-estimate/under-estimate force). We evaluated force estimation errors at 60 N at both the start and the end of testing to determine any between-group differences in perception of force following repeated tissue loading (factor: ‘time’). Given that participants with LBP might experience increased back soreness following repeated loading, which could feasibly influence the accuracy of their force estimation, this was important to consider.

**Figure 2:**
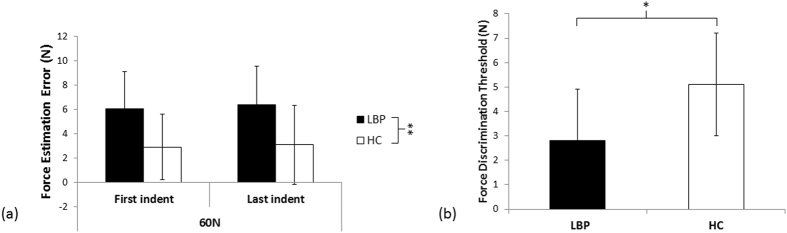
Study 1b results comparing healthy controls and people with chronic LBP and stiffness (**a**). Mean and standard error for force estimation error at 60 N for the first and last indentation (differed significantly between groups for both the first and last indentation at 60 N) (**b**). Mean and standard error for force discrimination threshold (differed significantly between groups). Abbreviations: LBP = low back pain; HC = healthy controls; *p < 0.05; **p < 0.001.

We found that while both groups consistently overestimated the force applied at both the start and end of testing, people with back pain had significantly higher force estimation errors than healthy controls (overestimating by 31% versus 15%; Fig. 2a). That is, the 2 × 2 repeated measures ANOVA found no main effect of time (F_1,28_ = 0.102, p = 0.75, partial η^2^ = 0.004), no interaction between time and group (F_1,28_ = 0.006, p = 0.94, partial η^2^ < 0.001), but a significant main effect of group (F_1,28_ = 19.1, p < 0.001, partial η^2^ = 0.405). This force overestimation was consistent at the individual level (Supplementary Figure S2). Intriguingly, in those with LBP and stiffness, the force overestimation error at 60 N was positively correlated with feelings of back stiffness (Spearman’s rho = 0.58, p = 0.025). That is, a stronger feeling of back stiffness was associated with greater force overestimation error.

As mentioned above, the forces were applied in pairs (two indentations 30 sec apart). In addition to magnitude estimation, participants were asked to identify whether the second indentation was the same or different than the first. Based on the answer, the force difference between the two indentations was adjusted (e.g., correct answer: decreasing from a 15 N force difference between indentations to an 11 N force difference between indentations). Repeating this procedure allowed us to identify the smallest difference in force that was reliably detected – the ‘force discrimination threshold’. We found that the groups differed in their ability to detect changes in force (Fig. 2b): people with LBP and stiffness detected a *smaller* difference in force between indentations of a pair, (smaller force discrimination threshold), than healthy controls did (t_2,28_ = 2.13, p = 0.04, d = 1.05). This enhanced ability to detect force differences was not because of differences in biomechanical properties of the spine: the change in objective stiffness that occurred at the discrimination threshold was no different between groups for either objective stiffness measure (AvgStiff: p = 0.29, TermStiff: p = 0.31).

### Study 2. Multisensory information modulates perception at the back

That feelings of back stiffness were unrelated to objective biomechanical measures of back stiffness, but did relate to overestimation of force applied to the spine, supports the idea that feeling stiff may represent a protective perceptual construct. As such, providing additional sensory input that enhances or reduces the need to protect the spine may have the ability to modulate perception at the back. Given clinical observations that sound is often paired with movement (e.g., hearing noise when a stiff joint or back moves), auditory input could feasibly modulate the need to protect the spine. Further, established multisensory interactions between somatosensory (‘feeling’) and auditory inputs suggests that modulation of perception is possible^53, 54^. Thus, Study 2 used established principles of multisensory integration to evaluate whether adding temporally congruent auditory information to the application of pressure to the spine would alter back perception. Participants (n = 20) estimated the amount of force received (expected forces between 50–70 N), but in all conditions, forces were applied at 60 N (equal chance of over-/under-estimation). We used force estimation as a surrogate for perceived stiffness to allow between-group comparison (healthy controls had negligible feelings of stiffness so could not rate it); this was validated as a surrogate prior to this experiment (see *Methods*).

**Figure 3:**
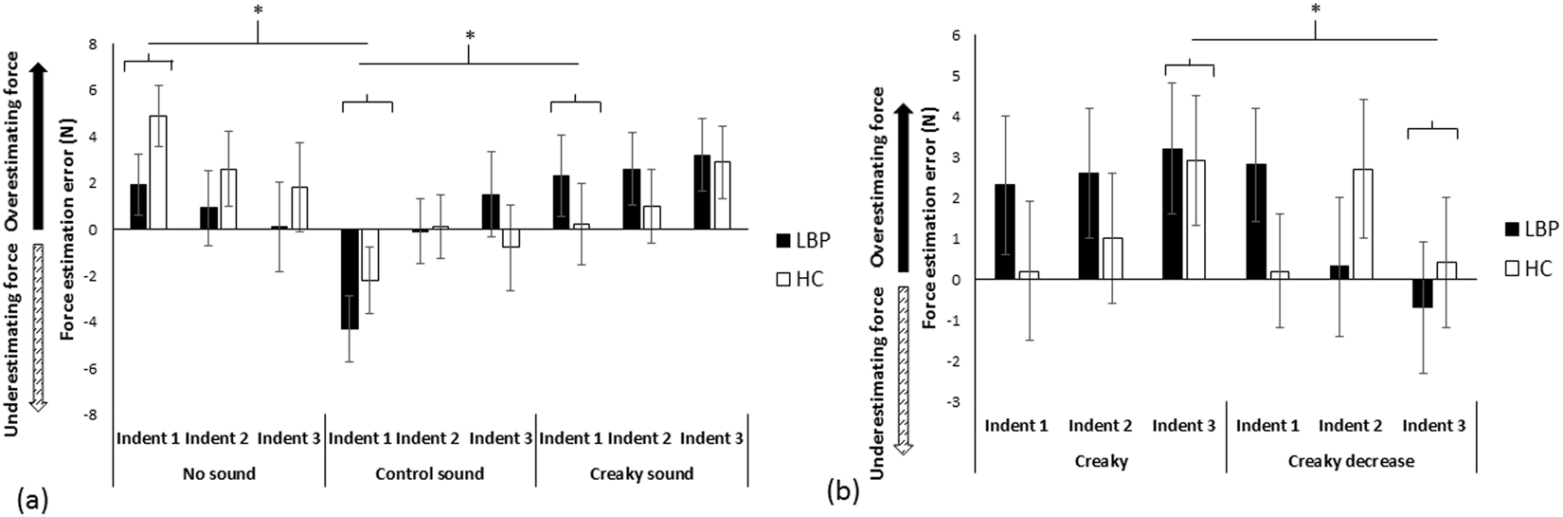
Study 2 results (**a**). Mean and standard error for force estimation error during the control, no-sound, and creaky sound conditions, compared between groups. A significant indent number x condition interaction occurred: significant differences were found between force estimation errors for Indent 1 between the creaky sound and control sound conditions and between the control sound and no-sound conditions; estimation errors did not differ between conditions for the other indentations, nor did they differ between groups. (**b**) Mean and standard error for force estimation error during the creaky sound and creaky decrease sound conditions, compared between groups. A significant indent number x condition interaction occurred: significant differences were found between force estimation errors for Indent 3 between the creaky sound and the creaky decrease conditions in posthoc analyses; estimation errors did not differ between conditions for the other indentations, nor did they differ between groups. Abbreviations: LBP = low back pain participants; HC = healthy controls; *p < 0.05.

First, we evaluated whether the effects on perception of the back (and force applied to it) were specific to the type of sound used – if so, this would provide compelling evidence that perceptual alterations were not merely due to the use of sound alone. We compared force estimation error (FEE) between groups during no-sound, creaky sound (“creaky door”), and control sound (“whoosh”) conditions (Supplementary Videos S1–S3). Each sound condition was repeated three times (i.e., over three consecutive indentations). We hypothesised that the creaky sound condition would significantly increase FEE compared with the other conditions and that this effect would be heightened in those with back pain and stiffness. We found that synchronising auditory information with the pressure applied to the spine influences back perception – there were significant differences in FEE over time (3 indentations) between the three sound conditions (Fig. 3a), but not between groups. That is, the RM ANOVA showed a main effect of Condition (F_1,18_ = 6.3, p = 0.005, partial η^2^ = 0.26), no effect of Group (F_1,18_ = 0.04, p = 0.84, partial η^2^ = 0.002), no effect of Indent number (F_2,36_ = 1.9, p = 0.16, partial η^2^ = 0.097), but a Condition x Indent number interaction (F_4,72_ = 3.2, p = 0.017, partial η^2^ = 0.15). No other interactions were significant (p = 0.20–0.59). *Post hoc* ANOVA tests revealed that the conditions differed in FEE for Indent 1 (F_2,38_ = 10.621, p < 0.0001, partial η^2^ = 0.36; Indent 2, p = 0.34; Indent 3, p = 0.19). For Indent 1, force estimations in the creaky sound condition (1.25 ± 5.5 N) were significantly higher than in the control sound condition (t_1,19_ = 3.28, p = 0.004, d = 0.74), where participants underestimated the force applied (−3.25 ± 4.6 N), but were no different from the no-sound condition (t_1,19_ = −1.40, p = 0.18, d = 0.32). The control sound condition significantly differed from the no-sound condition (t_1,19_ = 4.41, p < 0.0001, d = 0.99), where participants overestimated force applied (3.40 ± 4.3 N).

Second, we evaluated whether perception would be modified in both groups in line with change in the auditory input over time by comparing the creaky sound to a ‘creaky decrease’ condition (volume decreased over 3 indentations). We hypothesised that the creaky sound condition would increase FEE while the creaky decrease condition would decrease FEE with repeated indentations, and that this effect would be heightened in those with back pain and stiffness. Indeed, we observed opposite effects on force perception: whereas the constant creaky sound condition resulted in increasingly larger overestimation of applied force over each indent, the creaky decrease condition resulted in decreased force estimations over time (Fig. 3b). However, force estimations in both groups were similarly modulated. That is, the repeated measures ANOVA found a significant Condition x Indent number interaction (F_2,36_ = 3.40, p = 0.04, partial η^2^ = 0.16), no effect of Condition (F_1,18_ = 0.91, p = 0.35, partial η^2^ = 0.048), no effect of Indent number (F_2,36_ = 0.073, p = 0.93, partial η^2^ = 0.004), no main effect of Group (F_1,18_ = 0.11, p = 0.75, partial η^2^ = 0.006), and no other significant interactions (p = 0.12–0.48). *Post-hoc* paired comparisons showed that the conditions differed in FEE for the last indentation (t_1,19_ = 2.54, p = 0.02, d = 0.57): participants over-estimated force in the creaky condition (3.05 ± 4.8 N) relative to the creaky decrease condition, where force was under-estimated (−0.15 ± 4.9 N). No differences were found for the other indentations (Indent 1, p = 0.86; Indent 2, p = 0.85).

Importantly, there were no differences between Conditions in objective back stiffness (AvgStiff and TermStiff) as a function of Indent number or Group for either of the above comparisons (Supplementary Figure S3 and Table S2): perceptual alterations were not accompanied by changes in biomechanical stiffness. We also measured trunk muscle activity, i.e., muscle contractions insufficient to alter objective spinal stiffness measures, yet potentially relevant to altered perception. Overall, there were no consistent differences in muscle activity between conditions over time for any muscle group (Supplementary Table S3), suggesting that this was not the driver of altered perception.

## Discussion

A feeling of stiffness is an important predictor of disability, but the assumption that *feeling* stiff reflects actually *being* stiff has rarely been investigated. Our results provide compelling evidence that a *feeling* of back stiffness relates poorly to biomechanical measures of back stiffness. Consistent with our hypothesis, we show that a protective response exists in people who report feeling back pain and stiffness: they over-estimate applied force and are better than healthy controls at detecting any change in this force. Last, congruent auditory information applied with pressure to the spine modulates perception of the back, supporting the idea that a feeling of stiffness is a multisensory perceptual inference that serves bodily protection.

This research makes several important contributions to the existing literature. First, it provides empirical evidence that our conscious perception of stiffness is not derived solely from joint relevant sensory information: perceived and actual stiffness do not relate in our sample. In rheumatoid and osteoarthritis, the duration of feeling stiff does not relate to the degree of joint changes^24–26^, and our work suggests that the relationship is tenuous for stiffness intensity, in real-time, as well. Further, the lack of a difference in objective spinal stiffness between those with and without reported stiffness and LBP, is consistent with the idea that our bodily feelings reflect multimodal and evaluative processes, serving as behavioural drivers rather than markers of a biological or biomechanical state^27^. That is, people with back pain may feel stiff but it seems that this is not because they are objectively stiff; instead stiffness may be an effective perceptual mechanism to drive down movement and thus avoid provocation of nociception or injury. This also raises an important discussion of what people are describing when they refer to experiencing feelings of stiffness. While often described as a perceived resistance to movement, there is also suggestion that reports of feelings of stiffness may be a learned concept for what is actually a feeling of a lack of movement velocity^28^. It is interesting to consider that for some people, feelings of stiffness may also reflect more complex constructs such as fear of movement. Regardless, our results clearly show that the bodily experience of stiffness differs from the biomechanical tissue state.

Our results are in contrast to previous studies that found differences in objective spinal stiffness measures between those with and without acute/subacute LBP^29^ or neuropathic LBP (duration unspecified)^30^. In chronic LBP, treatment altered objective spinal stiffness measures and this alteration related to self-reported measures of disability^31^ – that is, actual back stiffness is changeable and may be important to some body-relevant perceptions. However, none of the previous studies evaluated whether the participants specifically had *feelings* of back stiffness, whereas we specifically recruited these participants. This makes direct comparison to our work difficult.

Our work also contributes to the knowledge of perceptual dysfunction in chronic conditions. There is consistent evidence of perceptual abnormalities in people with LBP: alterations in perceived shape of the back;^32^ reduced tactile acuity at the back;^32–34^ impaired motor imagery of the back;^35^ and impaired trunk voluntary motor control^36^. Our work suggests that this dysfunction extends to the perception of force applied to the back. Changes in the perception of touch have been linked to changes of receptive fields and response profiles of primary sensory cortex (S1) neurons^37^ – so-called cortical reorganisation^37, 38^ – raising the possibility that alterations in force perception might also represent cortical changes related to the back. That people with back pain are less accurate than healthy controls on a task requiring intact cortical proprioceptive representation supports this possibility^35^.

It is intriguing that people with LBP and stiffness could detect a smaller difference in force than healthy controls. It seems counter to findings of poor tactile acuity in people with LBP^32^. Superior force acuity yet inaccurate estimation of force magnitude, while seemingly incongruent, are together consistent with high attentional demands of pain^39^. Expecting pain can impair attentional disengagement^40, 41^ which may uniquely facilitate sustained attention to the back in the LBP group where indentations occurred over the painful area. Indeed, indentation evoked pain in those with LBP and stiffness but not in healthy controls. Expectations of pain and changes in attentional focus (e.g., spatial attention) are relevant because acuity in somatosensory (i.e., tactile) change detection has been shown to be improved by both^42, 43^ as has enhanced perception of threat^44^, which in other non-nociceptive paradigms increases protective reflexes^45^ – a finding that is largely consistent with protective force over-estimation seen here. Although the specific role of attention and expectation cannot be elucidated in our paradigm, that enhanced change detection extends to force magnitude in people with back pain is sensible and consistent with the ecological value associated with bodily protection. Given that this increased acuity in detecting differences in force was *not* accompanied by group differences in objective spinal stiffness suggests that it is more likely to reflect a top-down mechanism (e.g., attention-mediated), rather than a bottom-up one (e.g., superior mechanical detection).

While it is possible that features of the force discrimination task, such an indentation duration and response timing, played a role in the outcome seen, the likelihood of this is low. Regarding indentation duration, the indentor advanced at a constant velocity, meaning that differences in force targets (50 N vs 100 N) took slightly different time durations to reach. However, given our small range in force targets (50N-70N) this difference was less than a maximum of 1.5 seconds, and post-test questioning revealed that all participants based their force estimation judgements solely on the feel of the force on their back. None reported using time (indentation duration) to make these judgements. Second, regarding response timing, there was a 30 second interval between receiving the first and second indentation for the forced choice paradigm (response: same or different). This timing was essential given viscoelastic properties of the spine. Given that both groups received training to become comfortable with this task suggests that the impact of response timing is likely minimal.

Our findings of a heightened protective response in people that report back pain and stiffness raise the possibility that the mechanisms that subserve pain also contribute to the perception of stiffness. Such a possibility would be predicted by several relevant theoretical frameworks, for example: associative learning^46^ – pain and stiffness often go together; adaptation to pain model^47^ – pain-related muscle activation changes alter trunk stiffness^48, 49^ to prevent ongoing irritation of sensitive tissues; and the more recent link between motor effort and proprioception^50^ – painful movements are predicted to be more effortful [Tabor 2016, unpublished data], which in turn might make them feel stiffer. Together, these suggest that pain and stiffness likely compel similar behavioural responses that limit movement and thus re-injury. Such issues could be disentangled with further studies based on the current experimental paradigm. Indeed, further research is warranted to determine if this protective perceptual inference, e.g., a feeling of stiffness, results in protective behaviour such as movement avoidance in people with back pain and stiffness. That there was no difference between groups in force perceptual error over time suggests that perceptual differences are unlikely to be due to differences between groups in cognitive function (e.g., working memory capacity^51^).

This work provides crucial information to suggest how conscious perceptions are created, relevant to multisensory integration in chronic painful conditions. Our results suggest that auditory information is integrated with temporally congruent pressure information to create a perceptual experience and that this effect is not merely due to sound alone, but rather reflects the nature of this auditory information. Indeed, we found that the type and context of congruent sound had specific and varying effects on the modulation of perceptual error in force magnitude estimation. It is well-established that multisensory neurons in the superior colliculus receive information from auditory, visual and somatosensory inputs^52^. Further, substantial communication exists between the primary auditory cortex and the primary somatosensory cortex^53^, and auditory-somatosensory interactions exist in the early stages of cortical processing^54^. Last, integration of body-relevant information also occurs in the premotor^55^ and posterior parietal cortex^56, 57^ – the latter being an area that provides cognitive representations of our body and the space surrounding it.

Our discoveries support previous work that has shown that congruent auditory and tactile information can induce changes in the perceived properties of the hand: the so-called Marble Hand illusion^58^. Our work extends this finding by showing that the ability to integrate multimodal information and alter perception can also occur in a chronic pain state. Previous work has shown that touch and visual information can still be integrated in chronic pain: when a rubber hand illusion is applied people experience disownership of their painful limb in a similar manner to that observed in healthy controls^59^; the visual illusion of touch (using mirrored reflection of the good leg) improves tactile perception in the leg with chronic tactile deficits^60^. Our results extend these findings by confirming that this integration extends to audition and touch/pressure.

Our findings may have relevant implications for treatment. Improved outcomes in people with tendon pain are seen when exercises are paired with auditory cues^61^. Given the positive relationship between force estimation error and feelings of stiffness (the more stiff you feel – the greater the error), this raises the possibility that we may directly target feelings of stiffness with such auditory cues. Indeed, that we saw perceptual effects of sound in people with LBP and stiffness suggests that processes of multisensory integration are likely intact and thus may be a new and relevant treatment target. Finally, our work suggests that subjective and objective measures of stiffness are not proxies for one another, but likely capture different aspects of a similar domain. This raises the possibility that either self-perception or the physiological, objective aspects of back stiffness can be normal or abnormal. It follows then that exploring how various combinations of these states play out in intervention may reveal new mechanisms underlying back pain and/or approaches for its effective treatment.

## Conclusions

Here we show that a conscious experience of feeling stiff does not reflect true biomechanical back stiffness, but may rather represent a protective perceptual inference that may serve to reduce movement and re-injury. By showing that feelings of stiffness do not relate to biomechanical stiffness measures and that biomechanical measures of stiffness do not differ between those with and without feelings of stiffness, this suggests that information other than actual joint stiffness is influencing this perceptual inference. Although preliminary, our assertion that this interference is protective is substantiated by the heightened response to applied force in people with back pain and stiffness – they overestimate applied force magnitude but are more sensitive to detecting change. Last, synchronising auditory information in a way that conveys meaning to pressure applied to the back, modulates these protective responses in a specific manner dependent on the nature of the auditory information, and crucially, does so in the absence of changes to biomechanical properties of the spine. Future work is warranted to determine if this protective perceptual inference relates to protective behavioural responses (e.g., movement avoidance).

## Methods

Study 1a/1b recruited 30 participants (methods/results separated for clarity) providing 80% power to detect a moderate effect (f = 0.25) for force estimation and objective stiffness (2 groups, 2 measurement points, α = 0.05, repeated measure correlation = 0.60). Study 2 evaluated 20 participants from Study 1, providing 80% power to detect a small-to-moderate effect (f = 0.23) of auditory stimuli on force estimation (2 groups, minimum 6 measurement points, α = 0.05, repeated measure correlation = 0.60). These studies received ethical approval from the University of Alberta Human Research Ethics Board. All methods were carried out in compliance with the Declaration of Helsinki and all participants gave written informed consent. Informed consent was attained for publication of photographic and video images (Fig. 1a; Supplementary Videos S1–S3). All statistical tests were performed using IBM SPSS version 22.0 (New York, USA). Parametric analyses were used when raw data or transformed data met normality assumptions (inspection of probability plots, non-significant Shapiro-Wilk statistics); otherwise, non-parametric analyses were performed. All data are available from the corresponding author upon reasonable request.

### Study 1a

#### Participants

Fifteen people (27.1 ± 8.3 years, 4 female) with chronic (>3 months) non-specific LBP and self-reported stiffness, and 15 age- and gender-matched healthy volunteers without any current LBP/stiffness (28.9 ± 8.3 years, 5 female) were recruited. See Supplementary Table S4 for full eligibility criteria.

#### Equipment

A custom-designed, mechanical indentor applied rate-controlled anterior-posterior force to participants’ backs using a circular probe (40 mm^2^ head size) in a standardised manner while participants were prone (Fig. 1). This device is accurate (mean error of probe excursion versus gold standard: 0.00 ± 0.02mm)^62^, reliable^63, 64^, and has been used with asymptomatic and chronic LBP participants^21, 63, 64^. An electromechanical stepping motor (Dual Motion Motor, Waterbury, CT) drives probe movement, a compression-tension load cell (sensitivity of 20 mV/V; Entran, Fairfield, NJ) measures applied force, and a rotary encoder (Dual Motion Motor, Waterbury, CT) measures probe displacement. The probe advances at 2 mm/sec, pauses at a preload of 5 N (held 1 sec), then continues until the set target force is reached (held 1 sec), and then is withdrawn from the back.

#### General procedure

Participants completed baseline questionnaires (See Table 1). Then with participants positioned prone, the spinous process of the 3^rd^ lumbar vertebrae was manually identified using standardised procedures^65^ and the skin over L3 was marked. The probe was manually positioned at a standardised distance away from the back (10 mm; sufficient to avoid contact during participant inspiration). Three familiarisation indentations (target force = 30 N) accustomed participants to the procedure and to a standardised breathing pattern (a comfortable breath in and out, holding their breath in expiration for the duration of the indentation [~5 sec]). Following familiarisation, formal testing occurred where participants received a maximum of 20 indentations, ranging from 50–70 N.

### Primary outcome measures

#### Perceived stiffness

Participants rated their perceived current and average stiffness levels using 0–100 numerical rating scale, where 0 = no stiffness and 100 = worst stiffness imaginable.

#### Objective measures of spinal stiffness

During indentation, the applied force and the probe displacement were recorded by a customised Labview program (National Instruments 2015; Austin TX). Two measures of spinal stiffness (N/mm) were calculated: average stiffness (‘AvgStiff’: the slope of the force-displacement curve from10N to maximum force application; reflects tissue dynamics in response to force) and terminal stiffness (‘TermStiff’: average of the force/maximal displacement ratio over a one second time period when maximum force was held; reflects total bulk response)^63, 66^. These measures are reliable in both healthy and chronic LBP populations^63, 64^ and have been shown to predict response to manipulative therapy^21^ – a therapy of which one aim is to improve spinal mobility.

#### Secondary outcome – Pain intensity during indentation

After each indentation, participants rated the pain experienced on a 0–100 numerical rating scale (0 = no pain at all; 100 = most pain imaginable).

### Statistical analysis

The relationships between perceived back stiffness and objective spinal stiffness (at 55 N, 60 N and 70 N) in the LBP group were assessed using Spearman’s rho. For the healthy control group, perceived stiffness ratings were negligible.

Objective measures of spinal stiffness (AvgStiff and TermStiff) were analysed using a repeated measures Multivariate Analysis of Variance (RM MANOVA), comparing stiffness between groups for the first and last applied force at 60 N (within-subject: time; between-subject: group). If participants did not receive two indentations at 60 N, either two indentations at 59 N or 61 N were used (i.e., 60 N ± 1 N); this was matched between groups. A MANOVA was used to compare objective spinal stiffness measures at 55 N and 70 N between groups.

### Study 1b

Prior to formal testing (i.e., the 20 indentations described above), the same participants were trained in estimating force magnitude. Indentations forces at 50 N, 60 N, and 70 N were provided and participants were verbally advised of the magnitude of each impending force (“*This is what 50 N feels like”*). Participants were instructed to use these as reference points for future force estimations.

#### Procedure

Participants were told that they would receive forces of different magnitudes, but always between 50 N and 70 N, and asked to estimate, as accurately as possible (to within 1 N), the magnitude of force delivered (force magnitude estimation measure).

Indentations were delivered to participants’ backs in sets of two (‘force pair’), force estimations were provided for each indentation within the pair (indentations separated by 30 secs and force pairs separated by 1 min). Participants were also asked to indicate whether indentation 2’s force was the ‘same’ or different’ in magnitude to that of indentation 1 (force discrimination measure). Initial training included accustoming participants to this 30 second time interval.

A modified adaptive staircase procedure^23^ was used to increase and decrease the force difference between indentations of a pair. We started with a large force difference between indentations of a pair (e.g., 15 N); this difference decreased over subsequent indentation pairs until participants reported that they felt the forces to be of the same magnitude. Then the force difference between indentations of a pair was increased until participants reported the forces to be of different magnitudes. This process was repeated at least 3 times (maximum of 20 indentations), alternating between increasing and decreasing the force difference (Supplementary Methods S1 for full procedure).

### Primary outcomes

#### Force estimation error (FEE)

Error was calculated by subtracting the ‘actual’ force applied from the ‘estimated’ force magnitude (positive: overestimation of force; negative: underestimation of force). The primary outcome was the FEE at 60 N (applied force from 50–70 N, thus 60 N represents equal chance and magnitude of force over-/under-estimation).

#### Force discrimination threshold

This was defined as the smallest difference in force between indentations of a pair at which participants could detect a difference in force magnitude (e.g., Indent 1: 60 N, Indent 2: 65 N, 5 N force difference, response: ‘different’) but could not detect a difference in force between indentations of a pair when the force difference was 1 N smaller (e.g., Indent 1: 60 N, Indent 2: 64 N, 4 N force difference, response: ‘same’). In this example, the force discrimination threshold is 5 N.

#### Secondary outcome – Normalised objective stiffness changes

This was expressed as the absolute change in spinal stiffness at the force discrimination threshold as a proportion of that force difference: (spinal stiffness indent 2 - spinal stiffness indent 1)/(force indent 2 – force indent 1). This allowed us to determine whether between-group differences in force discrimination threshold may be due to differences in objective spinal stiffness changes.

### Statistical analysis

To determine if FEE differed between groups or as a function of time, a 2 × 2 RM ANOVA was used (within-subject: first and last 60 N force estimation error; between-subject: group). In participants not receiving two indentations at 60 N, 59 N or 61 N was used; this was matched between groups.

Independent t-tests investigated whether groups differed if force discrimination thresholds. Force discrimination threshold data required log_10_ transformation. For between group differences in normalised objective stiffness change, Mann Whitney U tests were used.

### Study 2

#### Participants

10 healthy control participants (23.4 ± 4.8 years, 3 female) and 10 chronic LBP and stiffness participants (25.7 ± 5.4 years, 4 female) were included in this study.

#### Procedure

Identical methodology was used for force application to the spine and for force estimation training. Participants were fitted with earbuds (Bose Pty Ltd., Newington, Australia) and protection ear muffs to minimise any errant noise. Participants completed four conditions, involving 3 indentations each, in a randomised order: (1) no sound; (2) creaky sound (‘creaky door noise’); (3) control sound (‘whoosh’); and (4) creaky decrease (volume reduced by 50% over each of the three indentations). All sounds were temporally paired to both the descent and withdrawal of the probe that applied force to the back. A customised Labview program delivered auditory stimuli, such that the timing of the sounds could be set to match the indentation (sound timing information covertly collected in Study 1). The investigator monitored indentation-sound timing in real-time (using earbuds) for all indentations.

Participants were instructed that they would receive forces anywhere between 50 and 70 N and asked to estimate, as accurately as possible, how much force they thought that they received. In reality, all forces applied were at 60 N (equal chance of over- and under-estimation).

#### Surface electromyography (sEMG)

Trunk muscle activity during indentation was measured using sEMG. Established skin preparation techniques were used before Ag/AgCl dipolar disposable electrodes (Ambu Blue Sensor M, London, Canada) were applied bilaterally to the external obliquus, the internal obliquus, and the erector spinae muscles using standardised locations (grounding electrode over the acromioclavicular joint). In a prone position (identical to testing), participants simultaneously contracted the truck musculature maximally (maximum voluntary contraction; MVC) three times.

#### Outcomes

FEE was calculated for each indentation (estimated magnitude minus 60 N) and used as a surrogate for feelings of stiffness to allow comparisons between groups (no feelings of stiffness in healthy controls). Using data from Study 1, we established that feelings of stiffness at baseline correlated significantly with FEE at 60 N (rho = 0.58, p = 0.025).

Objective spinal stiffness was measured in an identical manner to Study 1. For muscle activation, the raw sEMG signals were A/D converted (16 bit), full wave rectified and low pass filtered with a second order single pass Butterworth filter, using a cut-off frequency of 2.5 Hz. Customised Labview software (National Instruments 2015; Austin, TX), was used to calculate the average EMG amplitude over the indentation (marked by trigger points on the EMG file). The processed EMG data were then normalised to MVC amplitudes.

#### Statistics

To compare if the type of sound matters to force perception (FEE), a 3 (condition: no sound, control sound, creaky sound) × 3 (indent number: indent 1, indent 2, indent 3) × 2 (group) RM ANOVA was performed. To determine if the context of sound matters, a 2 (condition: creaky sound vs creaky decrease) × 3 (indent number: indent 1, indent 2, indent 3) × 2 (group) RM ANOVA was performed. When relevant, post hoc analyses using one-way RM ANOVAs and paired t-tests were completed (Holm-Bonferroni correction). Identical analyses were completed for objective spinal stiffness and for sEMG measures of muscle activation.

## Electronic supplementary material

Supplementary information

Video S1

Video S2

Video S3
