# Supplementary material for: Feeling stiffness in the back: a protective perceptual inference in chronic back pain
Source: Sci Rep. 2017 Aug 29;7:9681. doi: 10.1038/s41598-017-09429-1 (PMC5575135; doi:10.1038/s41598-017-09429-1)
Supplement: Supplementary file 1 — Supplementary information [file 41598_2017_9429_MOESM1_ESM.pdf]

Supplementary information

Manuscript Title:

Feeling stiffness in the back: a protective perceptual inference in chronic back pain

Authors: Tasha R Stanton, G Lorimer Moseley, Arnold Y L Wong, Gregory N Kawchuk

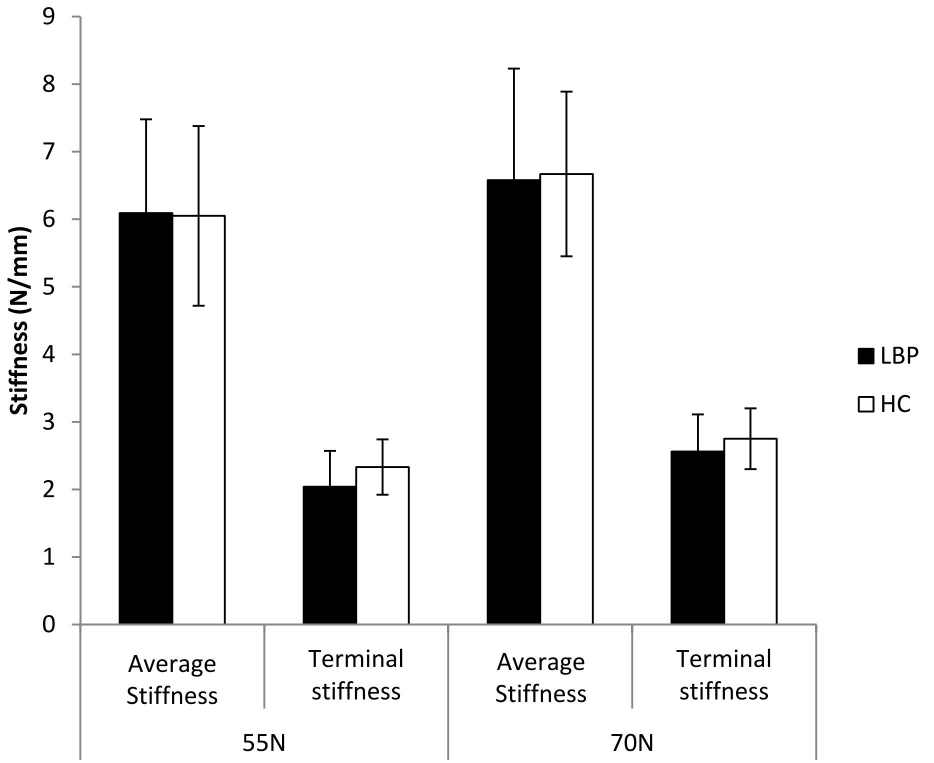

Figure S1. Mean and standard error for objective stiffness results, comparing between groups, for indentations applied at 55N and 70N. No differences were seen between people with low back pain and stiffness (LBP) and healthy controls (HC).

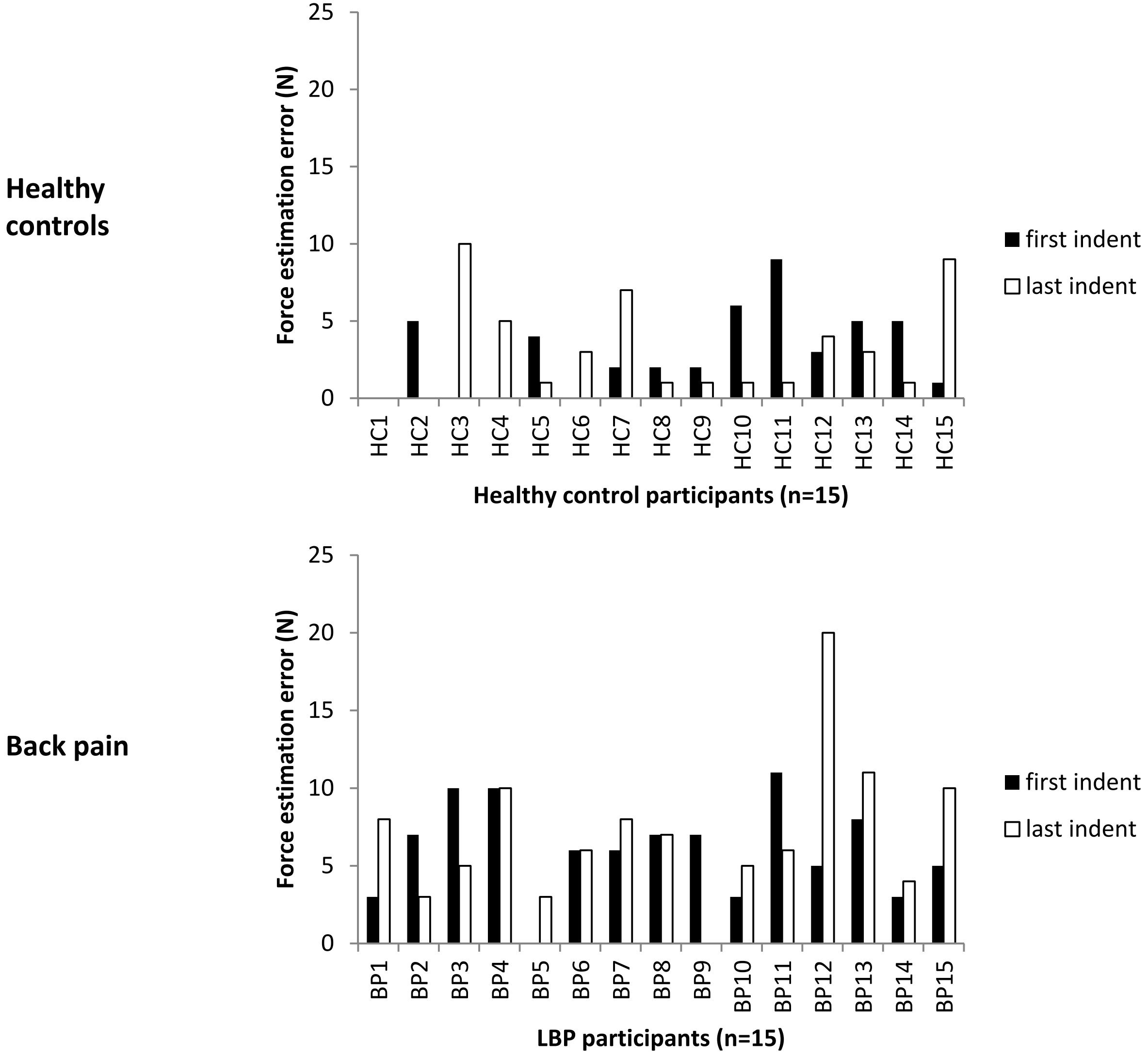

Figure S2. Individual participant force estimation errors for healthy controls and participants with chronic LBP and stiffness (positive = overestimating the force applied) at the first and last 60N indentation. Participants 11-15 (HC) and 10-11, 14 (BP) did not receive two indentations at 60N; instead results for two indentations at 59N or 61N were used (average matched between groups).

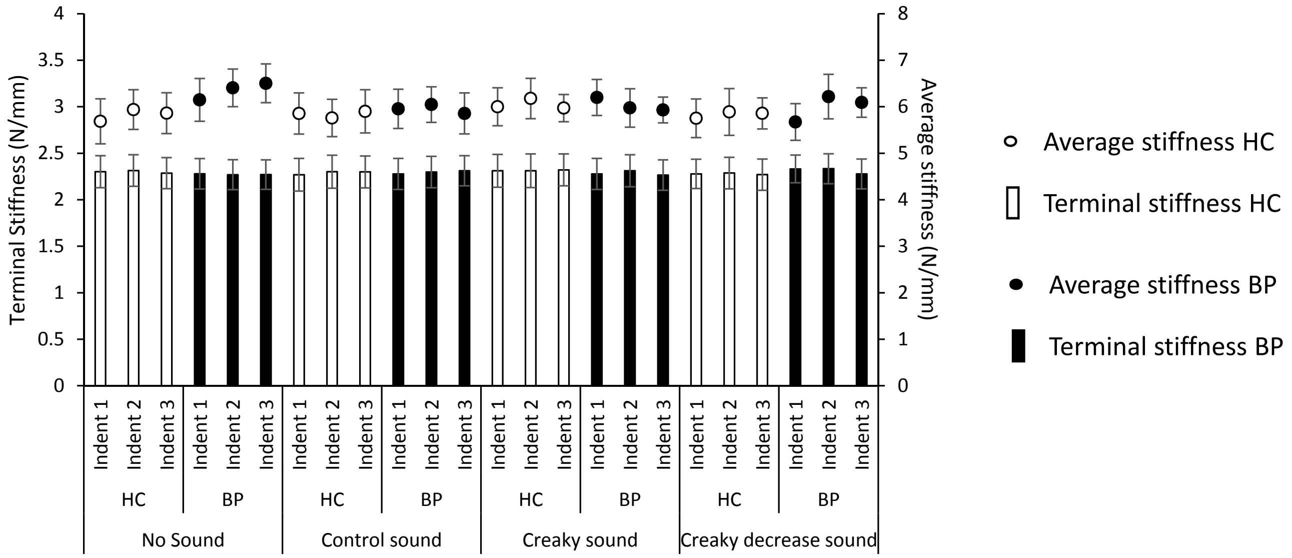

Figure S3. Mean and standard error for objective stiffness measures, compared between groups, for Study 2. Comparisons were made between the creaky condition versus control condition versus the no-sound condition and for the creaky condition versus creaky decrease condition. There were no differences between conditions, indent number, or groups for either stiffness measure. BP = chronic low back pain and stiffness; HC = healthy controls.

Table S1. Sensitivity analysis considering only those participants that received indentations at exactly 60N for Study 1a (objective stiffness measures) and for Study 1b (force estimation error). A repeated measures MANOVA was used to evaluate differences between groups in TermStiff and AvgStiff values over time (Study 1a). A repeated measure ANOVA was used to compare force estimation error between groups over time (Study 1b). The results were unchanged from analyses considering the full participant cohort.

**Effect**	**F-value**	**p-value**	**Partial η^2**
Study 1a: Repeated measures MANOVA for Objective stiffness at 60N: TermStiff AND AvgStiff			
Time x Group	$F_{2,19}$=0.518	0.60	0.052
Univariate results of MANOVA: TermStiff results			
Time	$F_{1,20}$ = 0.044	0.835	0.002
Time x Group	$F_{1,20}$ = 0.972	0.336	0.046
Group	$F_{1,20}$ = 0.155	0.698	0.008
Univariate results of MANOVA: AvgStiff results			
Time	$F_{1,20}$ = 0.410	0.529	0.020
Time x Group	$F_{1,20}$ = 0.019	0.891	0.001
Group	$F_{1,20}$ = 0.745	0.398	0.036
Study 1b: Repeated measure ANOVA for Force estimation error at 60N			
Time	$F_{1,20}$ = 0.526	0.476	0.026
Time x Group	$F_{1,20}$ = 0.013	0.911	0.001
Group	$F_{1,20}$ = 20.661	<0.001**	0.508

TermStiff = terminal stiffness; AvgStiff = average stiffness. **p-value significant at less than 0.001

Table S2. Terminal stiffness (TermStiff) and Average stiffness (AvgStiff) statistical results. Comparison 1: 3 (Condition: no sound, control, creaky) x 3 (Indent number: Indent 1, Indent 2, Indent 3) repeated measures ANOVA; Comparison 2: 2 (Condition: creaky vs creaky decrease) x 3 (Indent number: Indent 1, Indent 2, Indent 3) repeated measures ANOVA.

**Effect**	**F-value**	**p-value**	**Partial η^2**
**Comparison 1: No Sound versus Control Sound versus Creaky Sound**			
Objective stiffness: TermStiff results			
Condition	$F_{2,34} = 0.235$	0.792	0.014
Condition x Group	$F_{2,34} = 0.529$	0.594	0.030
Indent number	$F_{2,34} = 0.890$	0.420	0.050
Indent number x Group	$F_{2,34} = 0.016$	0.984	0.001
Condition x Indent number	$F_{4,68} = 1.730$	0.154	0.092
Condition x Indent number x Group	$F_{4,68} = 1.611$	0.431	0.051
Group	$F_{1,17} = 0.005$	0.943	0.000
Objective stiffness: AvgStiff results			
Condition	$F_{2,34} = 1.279$	0.291	0.070
Condition x Group	$F_{2,34} = 2.444$	0.102	0.126
Indent number	$F_{2,34} = 0.304$	0.740	0.018
Indent number x Group	$F_{2,34} = 0.079$	0.896	0.005
Condition x Indent number	$F_{4,68} = 0.613$	0.655	0.035
Condition x Indent number x Group	$F_{4,68} = 0.466$	0.760	0.027
Group	$F_{1,17} = 0.159$	0.695	0.009
**Comparison 2: Creaky Sound versus Creaky Decrease Sound**			
Objective stiffness: TermStiff results			
Condition	$F_{1,17} = 0.014$	0.908	0.001
Condition x Group	$F_{1,17} = 1.116$	0.306	0.062
Indent number	$F_{2,34} = 2.753$	0.078	0.139
Indent number x Group	$F_{2,34} = 2.145$	0.133	0.112
Condition x Indent number	$F_{2,34} = 0.395$	0.677	0.023
Condition x Indent number x Group	$F_{2,34} = 0.177$	0.839	0.010
Group	$F_{1,17} < 0.001$	0.989	0.000
Objective stiffness: AvgStiff results			
Condition	$F_{1,17} = 1.231$	0.283	0.068
Condition x Group	$F_{1,17} = 0.568$	0.461	0.032
Indent number	$F_{2,34} = 0.430$	0.654	0.025
Indent number x Group	$F_{2,34} = 0.007$	0.993	0.000

Condition x Indent number	$F_{2,34} = 1.404$	0.260	0.076
Condition x Indent number x Group	$F_{2,34} = 1.241$	0.310	0.067
Group	$F_{1,17} = 0.023$	0.881	0.001

Table S3: EMG results for activation levels expressed as a percentage of maximal voluntary contraction. Analysis 1: 3 (Condition: creaky vs no sound vs control sound) x 3 (Indent: 1, 2, 3) RM ANOVA; Analysis 2: 2 (Condition: creaky vs creaky decrease) x 3 (Indent: 1, 2, 3) RM ANOVA.

Comparisons	LEO	REO	LIO	RIO†	LES	RES
**Analysis 1. Creaky vs No Sound vs Control Sound**						
Condition	$F_{2,24} = 1.62$<br>$p = 0.218$<br>$\eta^2 = 0.119$	$F_{2,24} = 1.178$<br>$p = 0.325$<br>$\eta^2 = 0.089$	$F_{2,20} = 1.00$<br>$p = 0.386$<br>$\eta^2 = 0.091$		$F_{2,22} = 0.773$<br>$p = 0.474$<br>$\eta^2 = 0.066$	$F_{2,18} = 0.0815$<br>$p = 0.458$<br>$\eta^2 = 0.083$
Condition x Group	$F_{2,24} = 1.97$<br>$p = 0.16$<br>$\eta^2 = 0.141$	$F_{2,24} = 0.964$<br>$p = 0.396$<br>$\eta^2 = 0.074$	$F_{2,20} = 1.00$<br>$p = 0.386$<br>$\eta^2 = 0.091$		$F_{2,22} = 0.702$<br>$p = 0.506$<br>$\eta^2 = 0.060$	$F_{2,18} = 1.591$<br>$p = 0.231$<br>$\eta^2 = 0.150$
Indent	$F_{2,24} = 0.594$<br>$p = 0.56$<br>$\eta^2 = 0.047$	$F_{2,24} = 1.308$<br>$p = 0.289$<br>$\eta^2 = 0.098$	$F_{2,20} = 1.00$<br>$p = 0.386$<br>$\eta^2 = 0.091$		$F_{2,22} = 1.771$<br>$p = 0.194$<br>$\eta^2 = 0.139$	$F_{2,18} = 0.818$<br>$p = 0.457$<br>$\eta^2 = 0.083$
Indent x Group	$F_{2,24} = 1.727$<br>$p = 0.199$<br>$\eta^2 = 0.126$	$F_{2,24} = 2.945$<br>$p = 0.072$<br>$\eta^2 = 0.197$	$F_{2,20} = 1.00$<br>$p = 0.386$<br>$\eta^2 = 0.091$		$F_{2,22} = 1.983$<br>$p = 0.161$<br>$\eta^2 = 0.153$	$F_{2,18} = 0.818$<br>$p = 0.457$<br>$\eta^2 = 0.083$
Condition x Indent	$F_{4,48} = 1.319$<br>$p = 0.276$<br>$\eta^2 = 0.099$	$F_{4,48} = 2.156$<br>$p = 0.088$<br>$\eta^2 = 0.152$	$F_{4,40} = 1.00$<br>$p = 0.419$<br>$\eta^2 = 0.091$		$F_{4,44} = 1.422$<br>$p = 0.242$<br>$\eta^2 = 0.114$	$F_{4,36} = 0.818$<br>$p = 0.522$<br>$\eta^2 = 0.083$
Condition x Indent x Group	$F_{4,48} = 2.382$<br>$p = 0.064$<br>$\eta^2 = 0.166$	$F_{4,48} = 0.620$<br>$p = 0.185$<br>$\eta^2 = 0.119$	$F_{4,40} = 1.00$<br>$p = 0.419$<br>$\eta^2 = 0.091$		$F_{4,44} = 1.729$<br>$p = 0.161$<br>$\eta^2 = 0.136$	$F_{4,36} = 0.818$<br>$p = 0.522$<br>$\eta^2 = 0.083$
Group	$F_{1,12} = 0.685$<br>$p = 0.424$<br>$\eta^2 = 0.054$	$F_{1,12} = 0.017$<br>$p = 0.899$<br>$\eta^2 = 0.001$	$F_{1,10} = 1.930$<br>$p = 0.195$<br>$\eta^2 = 0.162$		$F_{1,11} = 0.472$<br>$p = 0.507$<br>$\eta^2 = 0.041$	$F_{1,9} = 0.029$<br>$p = 0.869$<br>$\eta^2 = 0.003$
**Analysis 2. Creaky vs Creaky decrease**						
Condition	$F_{1,12} = 2.43$<br>$p = 0.15$<br>$\eta^2 = 0.17$	$F_{1,12} = 1.698$<br>$p = 0.217$<br>$\eta^2 = 0.124$	$F_{1,11} = 0.846$<br>$p = 0.377$<br>$\eta^2 = 0.071$		$F_{1,11} = 0.448$<br>$p = 0.517$<br>$\eta^2 = 0.039$	$F_{1,9} = 0.075$<br>$p = 0.791$<br>$\eta^2 = 0.008$
Condition x Group	$F_{1,12} = 2.74$<br>$p = 0.12$<br>$\eta^2 = 0.19$	$F_{1,12} = 1.042$<br>$p = 0.327$<br>$\eta^2 = 0.08$	$F_{1,11} = 0.846$<br>$p = 0.377$<br>$\eta^2 = 0.071$		$F_{1,11} = 0.448$<br>$p = 0.517$<br>$\eta^2 = 0.039$	$F_{1,9} = 0.075$<br>$p = 0.791$<br>$\eta^2 = 0.008$
Indent	$F_{2,24} = 0.50$<br>$p = 0.61$<br>$\eta^2 = 0.04$	$F_{2,24} = 0.986$<br>$p = 0.388$<br>$\eta^2 = 0.076$	$F_{2,22} = 0.846$<br>$p = 0.443$<br>$\eta^2 = 0.071$		$F_{2,22} = 0.381$<br>$p = 0.687$<br>$\eta^2 = 0.034$	$F_{2,18} = 0.818$<br>$p = 0.457$<br>$\eta^2 = 0.083$
Indent x Group	$F_{2,24} = 0.103$<br>$p = 0.50$<br>$\eta^2 = 0.008$	$F_{2,24} = 4.298$<br>$p = 0.025$*<br>$\eta^2 = 0.264$	$F_{2,22} = 0.846$<br>$p = 0.443$<br>$\eta^2 = 0.071$		$F_{2,22} = 1.918$<br>$p = 0.171$<br>$\eta^2 = 0.149$	$F_{2,18} = 0.818$<br>$p = 0.457$<br>$\eta^2 = 0.083$
Condition x Indent	$F_{2,24} = 2.93$<br>$p = 0.073$<br>$\eta^2 = 0.196$	$F_{2,24} = 0.686$<br>$p = 0.513$<br>$\eta^2 = 0.054$	$F_{2,22} = 0.846$<br>$p = 0.443$<br>$\eta^2 = 0.071$		$F_{2,22} = 1.814$<br>$p = 0.186$<br>$\eta^2 = 0.142$	$F_{2,18} = 0.818$<br>$p = 0.457$<br>$\eta^2 = 0.083$
Condition x Indent x Group	$F_{2,24} = 1.24$<br>$p = 0.31$<br>$\eta^2 = 0.093$	$F_{2,24} = 0.242$<br>$p = 0.787$<br>$\eta^2 = 0.020$	$F_{2,22} = 0.846$<br>$p = 0.443$<br>$\eta^2 = 0.071$		$F_{2,22} = 1.814$<br>$p = 0.186$<br>$\eta^2 = 0.142$	$F_{2,18} = 0.818$<br>$p = 0.457$<br>$\eta^2 = 0.083$
Group	$F_{1,12} = 0.82$<br>$p = 0.38$<br>$\eta^2 = 0.064$	$F_{1,12} = 0.049$<br>$p = 0.828$<br>$\eta^2 = 0.004$	$F_{1,11} = 2.670$<br>$p = 0.130$<br>$\eta^2 = 0.195$		$F_{1,11} = 0.149$<br>$p = 0.707$<br>$\eta^2 = 0.013$	$F_{1,9} = 0.009$<br>$p = 0.927$<br>$\eta^2 = 0.001$

LEO = left external obliquus; REO = right external obliquus; LIO = left internal obliquus; RIO = right internal obliquus; LES = left erector spinae; RES = right erector spinae.†Analysis was unable to be run due to lack of variability in outcomes (error in SPSS because the values for each condition and for each indent were identical). * $p<0.05$

Table S4. Eligibility criteria for study participants.

	Back pain	Healthy controls	All participants
**Inclusion**	- Pain and stiffness in the low back area lasting at least 3 months	- No current low back pain or stiffness	- Aged between 18 and 60 years
**Exclusion**	- Previously undergone spinal surgery<br>- Suspected or confirmed nerve root involvement (defined as at least 2 of 4 congruent symptoms/signs: muscle weakness, dermatomal sensation changes, altered reflexes at the ankle/knee, positive straight leg raise)<br>- Suspected/confirmed malignancy as cause of back pain<br>- Current or previous (last 5 years) spinal fracture.	- Experienced a significant back pain episode in the past year (defined as back pain resulting in alterations to daily activities, including work)<br>- Experienced back pain with application of force to the spine	- Any disease/condition that prevented safe application of force to the spine (e.g., ankylosing spondylitis, severe spondylolisthesis, severe scoliosis, osteoporosis, rheumatoid arthritis, Type I diabetes mellitus, hyperparathyroidism, hyperthyroidism, currently taking muscle relaxants or disease modifying antirheumatic drugs)<br>- An inability to lie prone for at least 40 minutes<br>- An inability to tolerate indentations; and the inability to speak or read English

Supplementary Methods S1:

Modified adaptive staircase procedure to determine minimal force difference detection threshold

(maximum of 20 indentations)

1. Start with large difference in force between indentation pair (i.e., 15N). The difference in force between indentation pairs is reduced in increments of 4N (2N added to lowest force, 2N subtracted from upper force) until the person does not detect a difference (Response: 'same').

2. Increase the force difference for the indentation pair, this time using increments of 2N (1N added to lowest force, 1N subtracted from upper force). Continue until the person detects a difference between forces for the indentation pair (Response: 'different').

3. Decrease the force difference for the indentation pair, only changing by 1N (i.e. *either* adding 1N to lowest force *OR* subtracting 1N from upper force). Continue until the person no longer detects a difference between forces for the indentation pair (Response: 'same').

4. Increase the force difference for the indentation pair, only changing by 1N, and switch the order of the indentation pair, such that the larger force is given first. Continue until the person detects a difference between forces for the indentation pair (response: 'different').

5. Decrease the force difference for the indentation pair, only changing by 1N, until the person no longer detects a difference between forces for the indentation pair (Response: 'same').

Calculation of minimum force difference detection threshold: Determine all the occurrences when the participant detected both a difference between an indentation pair (response: 'different' when there was 5N force difference between indentations) and no difference between an indentation pair, when the force difference was 1N lower (response: 'same' when there was 4N force difference between indentations). Take an average of all the 'different' values that meet these criteria.

Scenario 1:

Force pair	Hypothetical Response (same or different)	Interpretation
1. 55/70	Different	Difference of 15N is detected
2. 57/68	Different	Difference of 11N is detected
3. 59/66	Different	Difference of 7N is detected
4. 61/64	Same	Difference of 3N is not detected
5. 60/65	Different	Difference of 5N is detected
6. 60/64	Same	Difference of 4N is not detected
7. 65/60	Different	Difference of 5N is detected
8. 64/60	Same	Difference of 4N is not detected
9. 65/60	Different	Difference of 5N is again detected
10. not needed		

Calculation:

1. 5N detected, 4N not detected

2. 5N detected, 4N not detected

Thus the minimum force difference that the person is able to detect is 5N.

Supplementary Methods S1:

Scenario 2:

**Force pair**	**Hypothetical Response (same or different)**	**Interpretation**
1. 55/70	Different	Difference of 15N is detected
2. 57/68	Different	Difference of 11N is detected
3. 59/66	Different	Difference of 7N is detected
4. 61/64	Same	Difference of 3N is not detected
5. 60/65	Same	Difference of 5N is not detected
6. 66/59	Different	Difference of 7N is detected
7. 66/60	Different	Difference of 6N is detected
8. 65/60	Different	Difference of 5N is detected
9. 64/60	Same	Difference of 4N is not detected
10. 60/65	Different	Difference of 5N is again detected; ensuring it wasn't a force order effect

Calculation:

1. 6N detected, 5N not detected

2. 5N detected, 4N not detected

Thus the minimum force difference detection threshold is 5.5N.

Video legends:

Video S1 – Example of the creaky sound paired with spinal indentation.

Video S2 – Example of the control sound paired with spinal indentation.

Video S3 – Example of the creaky decrease sound paired with three spinal indentations (decreases sequentially over each indentation).
